# Potential applications of artificial intelligence in image analysis in cornea diseases: a review

**DOI:** 10.1186/s40662-024-00376-3

**Published:** 2024-03-07

**Authors:** Kai Yuan Tey, Ezekiel Ze Ken Cheong, Marcus Ang

**Affiliations:** 1 Singapore National Eye Centre, 11 Third Hospital Ave, Singapore, 168751 Singapore; 2 Singapore Eye Research Institute, Singapore, Singapore; 3 Duke-NUS Medical School, Singapore, Singapore

**Keywords:** Artificial intelligence, Cornea, Machine learning, Deep learning, Anterior segment

## Abstract

Artificial intelligence (AI) is an emerging field which could make an intelligent healthcare model a reality and has been garnering traction in the field of medicine, with promising results. There have been recent developments in machine learning and/or deep learning algorithms for applications in ophthalmology—primarily for diabetic retinopathy, and age-related macular degeneration. However, AI research in the field of cornea diseases is relatively new. Algorithms have been described to assist clinicians in diagnosis or detection of cornea conditions such as keratoconus, infectious keratitis and dry eye disease. AI may also be used for segmentation and analysis of cornea imaging or tomography as an adjunctive tool. Despite the potential advantages that these new technologies offer, there are challenges that need to be addressed before they can be integrated into clinical practice. In this review, we aim to summarize current literature and provide an update regarding recent advances in AI technologies pertaining to corneal diseases, and its potential future application, in particular pertaining to image analysis.

**Supplementary Information:**

The online version contains supplementary material available at 10.1186/s40662-024-00376-3.

## Background

Artificial intelligence (AI) refers to a branch of computer science that enables development of artificial tools that mimics human cognitive processing function [1]. Whilst it was previously heavily utilized in the field of business, it has since infiltrated the field of medicine, given the increasing complexity of diseases and more importantly, presence of big datasets which allows us to utilize AI algorithms in assisting us to screen, diagnose and prognosticate diseases. This has also allowed us to gradually move towards an “intelligent healthcare” structure as there is mounting evidence of possibility in applying AI-driven algorithms in clinical practice.

Currently, there are two main subsystems in the realm of AI research that is being utilized and have been heavily researched on within the field of medicine—machine learning (ML) and deep learning (DL), of which the latter is a subtype of ML [2]. ML refers to an AI tool that, with programmed algorithms, is able to analyze and learn from a trained dataset, and to apply the process in making a similar decision process as the input data [2]. ML could be further categorized into 1) supervised ML, such as support vector machine (SVM) and random forest (RF) classifier, 2) unsupervised ML, 3) semi-supervised ML and 4) reinforcement ML [3]. A RF classifier works through the construction of multiple decision trees and the output/conclusion is determinded by the class derived by most decision trees. A SVM, in addition to linear classification, is able to find a hyperplane in a dimensional space where there multiple number of features, that provides the maximum margin.

**Fig. 1:**
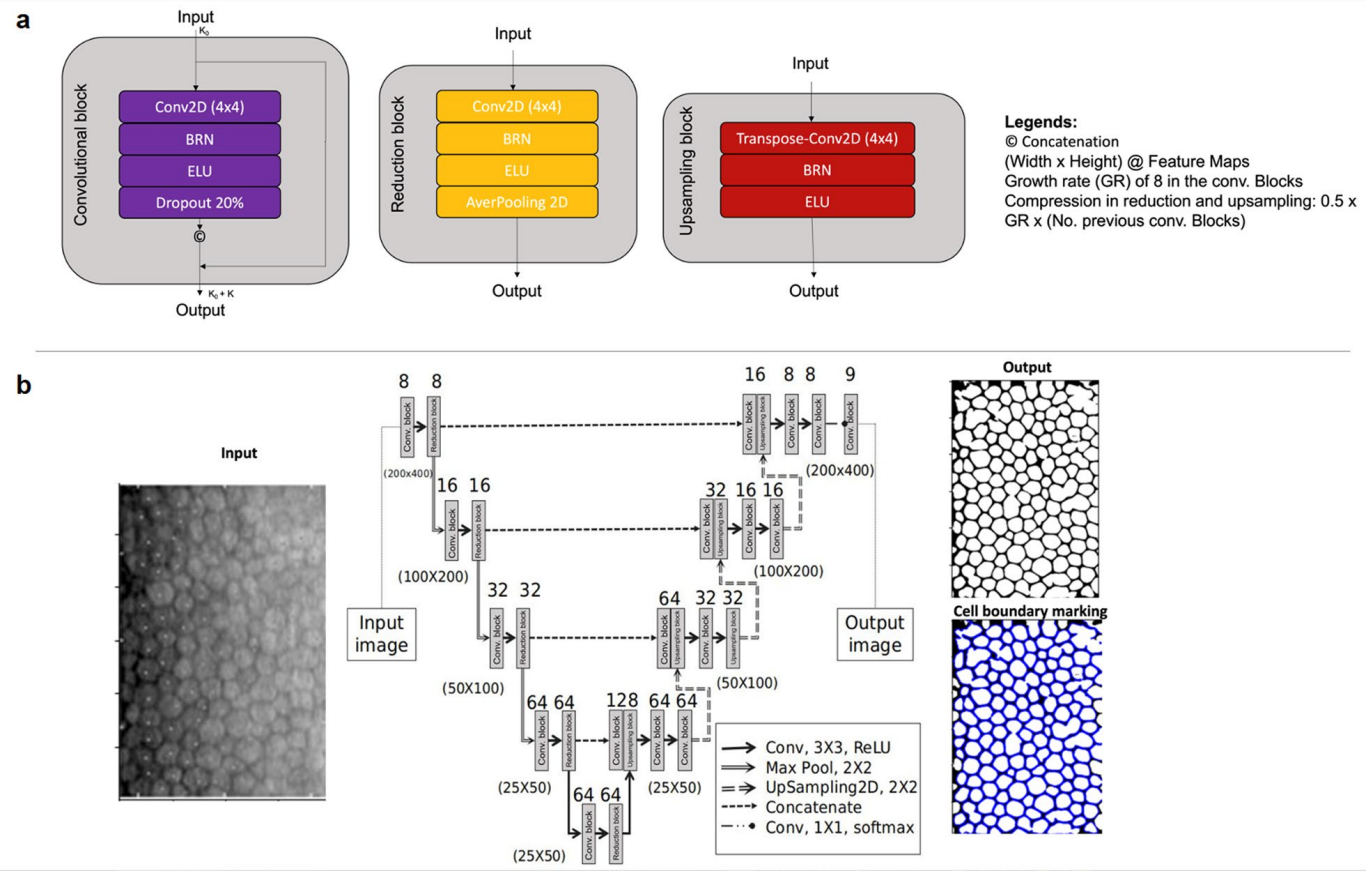
Schematic overview of a deep learning (DL) network, in specific convolutional neural network (CNN). **a** Demonstrated various blocks found in CNN, in specific, convolutional, up sampling and reduction blocks, which are sliding window filters that execute operations on images. **b** demonstrated the flow of CNN, in particular in this figure, the CNN algorithm would extract features from a raw data input i.e. specular microscopy image, which will then automatically segment the image as a target, producing an edge image as an output, which would then undergo a post-processing directly to produce the final binary segmented image with cell boundary marking

DL, on the other hand, whilst often used interchangeably with ML, is a subset of ML which utilizes a neural network consisting of layers of neurons [4]. The aim of the DL network is to replicate the complex neural network of a human brain as the layers of neurons communicate amongst each other for input and output, hence allowing progressive extraction of higher-level data output from raw data input [4]. In essence, neural network-based algorithm is able to extract features from input data, and identify “hidden pattern” to reach a conclusion through a decision process, similar to the human cognitive process, which is known as the “AI black box”. In contrast to other forms of ML, the nature of DL opposes the need to extract data manually, which is less labor intensive. The main subtypes are convolutional neural network (CNN), which includes but is not limited to U-Net, VGG, MobileNet, and LeNet, and recurrent neural network (RNN) which includes but is not limited to one-to-one RNN, one-to-many RNN, many-to-one RNN and many-to-many RNN. An example of CNN can be seen in Fig. 1 which demonstrates the layers of neuronal networks to segment endothelial cells based on specular microscopy.

While AI has shown promising applications in other medical specialties such as radiology, pathology, and cardiology, there is an increasing interest in developing reliable and effective tools in the field of ophthalmology. Given the nature of ophthalmology, there exists big datasets in the form of ophthalmic images obtained through various form of modalities, which provide a good avenue to adopt AI-driven algorithms into clinical practice. This paper aims to review the current state of the AI application in the field of cornea, in particular, pertaining to image analysis.

## Main text

### Literature search

We conducted a literature search via PUBMED and MEDLINE database for articles written in English until 9th August 2023, with the following medical subject headings: “Artificial Intelligence”, “Cornea”, “Anterior Segment”, “Deep learning” and “Machine learning”. Bibliographies of included articles were manually screened to identify further relevant studies. Publications that were not in English were excluded from this review. In total, 281 studies were identified and screened, with a total of 107 studies included in this review.

### Artificial intelligence in cornea – early detection and screening

In line with the current climate of ophthalmology AI research, cornea-related diseases also rely heavily on big data and analysis of images. Diagnosis and management of cornea diseases often rely on various imaging modalities such as slit-lamp photography, corneal topography, anterior-segment optical coherence tomography (AS-OCT), specular microscopy, in vivo corneal confocal microscopy (IVCM). These modalities could be utilized in the training of AI-algorithms to assist with achieving an automatic process in diagnosing or screening of corneal conditions. The list of studies that are related to detection and screening for keratoconus (KC), infectious keratitis (IK), pterygium, and dry eye diseases (DEDs) are found in Table 1 (KC and IK) and Table 2** (**pterygium and DEDs**)**. Owing to its large quantity, only KC-related studies published from 2022 to 2023 were included in Table 1, and studies published earlier than the indicated time period can be found in Additional file 1: Table S1.

**Table 1:** A summary table of artificial intelligence (AI) application in the diagnosis of keratoconus and dry eye diseases, in reverse chronological order

Year	Authors	Imaging modality	Sample size (eyes)	Study population	Outcome measures	AI algorithms	Diagnostic performance	Validation method
Keratoconus								
2023	Lu et al. [15]	Pentacam, SD-OCT, APT	599	Healthy, FF, early, advanced KC eyes	KC detection	RF/CNN	AUC: 0.801–0.902	Hold-out validation
2023	Kundu et al. [111]	AS-OCT	1125	Healthy, VAE and KC eyes	KC detection	RF	AUC: 0.994–0.976, Acc: 95.5%–95.6% Sens: 71.5%–98.5%, Precis: 91.2%–92.7%	Hold-out validation
2022	Cohen et al. [112]	Galilei	8526	Healthy, suspect and KC eyes	KC detection	RF	AUC: 0.964–0.969, Acc: 90.2%–91.5% Sens: 94.2%–94.7%, Spec: 89.6%–89.8%	Hold-out validation
2022	Almeida Jr et al. [113]	Pentacam	2893	Healthy, VAE and KC eyes	KC detection	BESTi MLRA	AUC: 0.91, Sens: 86.02% Spec: 83.97%	Hold-out validation
2022	Reddy et al. [114]	Oculyzer	1331	Healthy and KC eyes	Prediction of latent progression of KC	CNN	11.1 months earlier progression than KP (*P* < 0.001)	Hold-out validation
2022	Gao et al. [115]	Pentacam	208	Healthy, subclinical and KC eyes	Subclinical and KC detection	KeratoScreen ANN	Sens: 93.9%–97.6% Precis: 95.1%–96.1%	Hold-out validation
2022	Xu et al. [116]	Pentacam	1108	Healthy, VAE and KC eyes	Detection of healthy eye in VAE	KerNet CNN	Acc: 94.67% AUC: 0.985	Hold-out validation
2022	Gairola et al. [117]	SmartKC	57	Healthy and KC eyes	KC detection	CNN	Sens: 91.3% Spec: 94.2%	Hold-out validation
2022	Lu et al. [65]	SD-OCT, APT	622	Healthy, FF, early, advanced KC eyes	KC detection	RF/CNN	AUC: 0.99, Sens: 75% Spec: 94.74%	Hold-out validation
2022	Subramaniam et al. [118]	Pentacam	900	Healthy, subclinical and KC eyes	KC detection and grading	PSO, GoogLeNet CNN	Acc: 95.9%, Spec: 97.0% Sens: 94.1%	Hold-out validation
2022	Mohammadpour et al. [12]	Pentacam, Sirius, OPD-Scan III Corneal Navigator	200	Healthy, subclinical and KC eyes	KC detection	RF	Subclinical KC – Acc: 88.7%, Sens: 84.6%, Spec: 90.0% KC – Acc: 91.2%, Sens: 80.0%, Spec: 96.6% (Based on Sirius Phoenix)	N.A
Dry eye diseases								
2023	Shimizu et al. [54]	ASV	158	Healthy and DED eyes	DED grading based on TBUT	ImageNet-22 k CNN	Acc: 78.9%, AUC: 0.877 Sens: 77.8%, Spec: 85.7%	Hold-out validation
2023	Abdelmotaal et al. [52]	ASV	244	Healthy and DED eyes	DED detection	CNN	AUC: 0.98	Hold-out validation
2022	Fineide et al. [51]	ASV	431	Patients with DED	DED grading based on TBUT	RF	Sens: 99.8%, Precis: 99.8% Acc: 99.8%	Cross validation
2022	Edorh et al. [119]	AS-OCT	118	Healthy and DED eyes	Epithelial changes as a marker of DED	RF	Sens: 86.4% Spec: 91.7%	N.A
2021	Chase et al. [44]	AS-OCT	151	Healthy and DED eyes	DED detection	VGG19 CNN	Acc: 84.62%, Sens: 86.36% Spec: 82.35%	Hold-out validation
2021	Elsawy et al. [120]	AS-OCT	879	Healthy and various anterior segment eye diseases	DED detection	VGG19 CNN	AUC: 0.90–0.99	Hold-out validation
2020	Maruoka et al. [62]	HRT-3 confocal microscopy	221	Healthy and obstructive MGD eyes	Obstructive MGD detection	Multiple CNNs	AUC: 0.96, Sens: 94.2% Spec: 82.1%	Hold-out validation
2020	da Cruz et al. [56]	Doane interferometer	106	VOPTICAL_GCU database of tear film images	Classification of tear film lipid layer	SVM, RF, NBC, MLP, RBFNetwork, random tree	Acc: 97.54%, AUC: 0.99 κ: 0.96	Cross validation
2020	Stegmann et al. [121]	AS-OCT	6658	Healthy eye images	Tear meniscus segmentation	TBSA CNN	Sens: 96.4%, Spec: 99.9% Jaccard index: 93.2%	Cross validation
2019	Wang et al. [122]	Keratograph 5 M	209	Healthy and DED eyes	Segmentation of meibomian gland, grading of meiboscore	CNN	Acc: 95.4%–97.6% IoU: 66.7%–95.5%	Hold-out validation
2018	Arita et al. [55]	DR-1α tear interferometer	100	Healthy and DED eyes	DED detection and grading	Interferometric movies	κ: 0.76 Acc: 76.2%–95.4%	N.A

*Acc* = accuracy; *ANN* = artificial neural network; *APT* = air puff tonometry; *AS-OCT* = anterior-segment optical coherence tomography; *ASV* = anterior segment videography; *AUC* = area under curve; *CNN* = convolutional neural network; *DED* = dry eye disease; *FF* = forme fruste keratoconus;* IoU* = intersection over union; *IVCM* = in vivo confocal microscopy; *κ* = kappa index; *KC* = keratoconus; *KP* = keratometric progression; *MGD* = meibomian gland disease; *MLP* = multilayer perceptron; *N.A.* = not available; *NBC* = naïve Bayes classifier; *Precis* = precision; *RF* = random forest; *SD-OCT* = spectral-domain optical coherence tomography; *Sens* = sensitivity; *Spec* = specificity; *SVM* = support vector machines; *TBSA* = threshold based algorithm; *TBUT* = tear breakup time; *VAE* = very asymmetric eyes (fellow to KC eyes)

Jaacard index is a statistical analysis of how similar two sample sets are

**Table 2:** A summary table of artificial intelligence (AI) applications in the diagnosis of pterygium and infectious keratitis, in reverse chronological order

Year	Authors	Imaging modality	Sample size (eyes)	Study population	Outcome measures	AI algorithms	Diagnostic performance	Validation method
Pterygium								
2023	Liu et al. [36]	ASP	20,987	Dataset of smartphone and slit-lamp eye images	Detection and segmentation of pterygium	SA-CNN, SRU-Net	Detection Acc: 95.24% Segment Acc: 89.81%, Sens: 87.09% Spec: 96.68%, AUC: 0.9295	Hold-out validation
2022	Wan et al. [123]	ASP	489	Healthy and pterygium eyes	Segmentation and measuring of pterygium	U-Net + + CNN	Dice: 0.902–0.962 κ: 0.918	Hold-out validation
2022	Fang et al. [42]	ASP	6311	SEED eye images; healthy and pterygium eyes	Detection of pterygium	CNN	AUC: 0.995, Sens: 98.6% Spec: 99.0%	Cross validation
2022	Hung et al. [45]	ASP	237	Healthy and pterygium eyes	Detection of pterygium, and prediction of recurrence post-excision	Deep learning network	Pterygium detection – Spec: 91.7% – 100%, Sens: 80% – 91.7% Prediction of recurrence – Spec: 81.8%, Sens: 66.7%	Hold-out validation
2022	Zhu et al. [124]	ASP	300	Healthy and pterygium eyes	Detection and segmentation of pterygium	Multiple CNNs	Acc: 99.0%, κ: 0.98 Sens: 98.7%, Spec: 99.3%	Hold-out validation
2021	Jais et al. [125]	BCVA	93	Pterygium patients undergoing surgery	Prediction of BCVA improvement post-pterygium surgery	SVM, NBC, decision tree, logistic regression	Acc: 94.44%, Spec: 100% Sens: 92.14%	Cross validation
2019	Zulkifley et al. [126]	ASP	120	Healthy and pterygium eyes	Detection of pterygium	CNN	Acc: 81.1%, Sens: 95.0% Spec: 98.3%	Cross validation
2019	Lopez et al. [127]	ASP	3017	Healthy and pterygium eyes	Detection of pterygium	CNN	AUC: 0.99, Acc: 93.5% Sens: 88.2%	N.A
Infectious keratitis								
2023	Essalat et al. [37]	IVCM	1001	IVCM-Keratitis dataset images	IK detection	Densenet161 CNN	Acc: 93.55%, Precis: 92.52% Sens: 94.77%	Cross validation
2023	Liang et al. [36]	IVCM	7278	Dataset of FK eye images	FK detection	GoogLeNet, VGGNet CNNs	Acc: 97.73%, Sens: 97.02% Spec: 98.54%	Hold-out validation
2023	Wei et al. [128]	ASP	420	FK, BK, AK and VK eyes	FK detection and discriminating	Binary logistic regression, decision tree classification, RF	AUC: 0.859–0.916	Cross validation
2022	Natarajan et al. [31]	ASP	285	HSV VK and BK eyes	VK detection	DenseNet CNN	Acc: 72%, AUC: 0.73 Sens: 69.6%, Spec: 76.5%	Hold-out validation
2022	Redd et al. [129]	ASP	980	FK and BK eyes	FK discriminating from BK	MobileNet CNN	AUC: 0.86	Cross validation
2022	Ghosh et al. [35]	ASP	194	FK and BK eyes	FK discriminating from BK	VGG19, ResNet50, DenseNet121 CNNs	Acc: 68.0%–78.0% AUC: 0.60–0.86	Hold-out validation
2021	Kuo et al. [130]	ASP	1512	Clinically suspected IK eye images	BK detection	Multiple CNNs	Sens: 74% Spec: 64%	Cross validation
2021	Wang et al. [28]	ASP	6073	Healthy, FK, BK and HSV VK eye images	FK, BK, HSV VK detection	InceptionV3	κ: 0.538 – 0.913 AUC: 0.860 – 0.959	Hold-out validation
2021	Koyama et al. [131]	ASP	4306	BK, AK, HSV VK eye images	BK, AK, HSV VK detection	Gradient boosting decision tree	Acc: 92.3%–97.9% AUC: 0.946–0.995	Cross validation
2021	Li et al. [132]	ASP	6925	Healthy and keratitis eye images	Keratitis detection	DenseNet121 CNN	AUC: 0.96	Hold-out validation
2020	Kuo et al. [133]	ASP	288	IK confirmed eyes	FK detection among IK	DenseNet CNN	Sens: 71%, Spec: 68% Acc: 70%	Cross validation
2020	Liu et al. [134]	HRT-3 confocal microscopy	1213	Healthy and FK eyes	FK detection	CNN	Acc: 100%, Sens: 99.9% Spec: 100%	N.A
2018	Wu et al. [135]	HRT-3 confocal microscopy	378	Healthy and FK eyes	Hyphae detection	KNN, SVM, linear regression, decision tree	AUC: 0.86–0.98 Acc: 81.7%–99.1% Sens: 78.5%–98.5%	Cross validation

*Acc* = accuracy; *AK* = acanthamoeba keratitis; *ASP* = anterior-segment photography; *AUC* = area under curve; *BCVA* = best-corrected visual acuity; *BK* = bacterial keratitis; *CNN* = convolutional neural network; *FK* = fungal keratitis; *HSV* = herpes simplex virus; *IK* = infectious keratitis; *IVCM* = in vivo confocal microscopy; *κ* = kappa index; *KNN* = K-nearest neighbour; *ML* = machine learning; *NBC* = naïve Bayes classifier; *N.A*. = not available; *Precis* = precision; *RF* = random forest; *SA-CNN* = self-attention convolutional neural network; *SEED* = Singapore Epidemiology of Eye Diseases; *Sens* = sensitivity; *Spec* = specificity; *SVM* = support vector machine; *VK* = viral keratitis

### Keratoconus

One of the key features of AI-driven tools in cornea diseases is its potential in assisting clinicians in screening, diagnosis and/or prognostication of cornea conditions of which a key example is KC. KC refers to a non-inflammatory condition involving both corneas asymmetrically, and was found to affect between 0.2 to 4790 per 100,000 persons [5]. Early detection could allow surgeons to monitor disease progression and if necessary, to intervene appropriately to halt progression through corneal cross-linking [6]. KC also presents as the leading cause of ectasia post-laser vision correction and hence ideally should be detected pre-treatment [7, 8]. Therefore, identification of subclinical keratoconus (SKC) is important in screening for suitable candidates for refractive surgery. In recent years, diagnosis of KC could be made more easily and accurately with widespread use of corneal topography, AS-OCT, and Scheimpflug imaging [5]. While identification of KC could be done relatively easily with prominent clinical signs and objective data such as biomicroscopic and keratometric data, it can be challenging for clinicians to identify early KC which may lack specific signs. Early KC can be further sub-categorized into SKC, KC suspect (KCS), forme fruste KC (FFKC) [9].

The idea of application of AI algorithm in the screening of KC was first described in 1997 by Smolek et al. who used a neural network to analyze corneal topography images and found that neural network was able to distinguish KC from KCS [10]. Recently, application of AI in assisting KC screening and diagnosis has garnered increasing attention. Kuo et al. trained multiple CNN algorithms to screen for KC using corneal topography images of 354 eyes (170 KC, 28 SKC, and 156 normal eyes) without manual segmentation [11]. Overall, the study group found that sensitivity and specificity were over 90% for all models—in particular, the specificity of ResNet152 was 97% (AUC = 0.99), which suggested its potential to be used for KC screening owing to its low false-negative rate. Its drawback, however, was its low accuracy of discriminating SKC from healthy eyes (accuracy of 28.5%) [11]. Mohammadpour et al. instead compared RF classifier models based on different inputs—(1) Pentacam Belin/Ambrosio enhanced ectasia total deviation value and Topographic Keratoconus Classification, (2) Sirius Phoenix, and (3) OPD-Scan III Cornea Navigator in identifying healthy eyes, SKC and KC [12]. They found that the most robust method would be using Sirius Phoenix with an accuracy for differentiating KC (91.24%) and SKC (88.68%) [12].

Cao et al. utilized a RF classifier with reduced dimensionality representation of comprehensive Scheimpflug tomography parameters and was able to identify SKC from healthy eyes with a high sensitivity of 97% (AUC = 0.98) [13]. Feng et al. reported a trained CNN named KerNet utilizing raw data obtained by Scheimpflug tomography, and were able to achieve high performances on detecting KC (AUC = 0.99) and SKC (AUC = 0.97) [14]. Lu et al. described a method of analyzing multiple imaging modalities such as spectral-domain optical coherence tomography (SD-OCT), air puff tonometry, and Scheimpflug tomography using RF algorithm and neural network [15, 16]. The group used 599 eyes for training and found that overall, a high AUC was attained when RF was applied to select features from SD-OCT and air puff tonometry (AUC = 0.91) [15, 16].

Al-Timemy et al. also proposed a method to improve the detection of KC through ensemble of deep transfer learning (EDTL) based on cornea topographic maps. They utilized four pre-trained network—SqueezeNet, AlexNet, ShuffleNet, and MobileNet-v2, and a classifier system [17]. Their EDTL would then consider the output from all networks to obtain a conclusion. Through this complex and robust method, they were able to achieve an accuracy of 98.3% compared to relying on individual network (92.2% with just SqueezeNet, 93.1% for AlexNet) [17].

An additional benefit of using an AI algorithm is its potential use of identifying a FFKC from a normal eye which may otherwise be clinically difficult and hence allowing closer monitoring and earlier intervention if needed. Unsupervised ML was used to classify KC by analyzing SS-OCT images, along with the corresponding corneal topography, elevation and pachymetry parameters and ectasia severity index [18]. The algorithm was able to achieve a sensitivity of 96.0% and specificity of 97.4% in discriminating KC from normal eyes [18]. The same algorithm also succeeded in highlighting a portion of normal eyes as mild KC, which was postulated to be FFKC and hence warranted further attention from clinicians. In an AI-based model, combination of various modalities could also prove to be useful in the detection of KC when compared to using just one modality. This was demonstrated by a meta-analysis done by Hashemi et al. which revealed that a combination of Scheimpflug and Placido corneal imaging methods would provide high diagnostic accuracy for early KC detection when compared to a single modality on its own [19].

In addition to KC screening, effort has been put into developing tools in screening for progression and classification of severity in KC management. CNNs were trained to detect an increase in anterior curvature [maximum keratometry (Kmax)] on Scheimpflug tomography images to classify them into either the progression or non-progression group. The corresponding changes in other Scheimpflug tomography parameters were also incorporated to train and cross-validate the AI model. The AI model was able to achieve a sensitivity of 86% and specificity of 82% (AUC = 0.90) in detecting a progression [20]. The AI models were also able to predict concomitant progression in other Scheimpflug tomography parameters i.e., global progression amongst 60%–62% of actual progression eyes. This may be useful in screening and monitoring of known patients with KC and hence allow early detection and recall of patients as they may require early intervention.

In a prospective study done by Kundu et al., using a RF classifier, they were able to predict if an eye would progress (AUC = 0.812). The classifier utilised a series of clinical and ocular surface risk factors which included habit of eye rubbing, duration of indoor activity, habit of using lubricants and immunomodular topical medications, duration of computer use, hormonal disturbances, use of hand sanitiser, biomarkers such as vitamins D and B_12_, and immunoglobulin E [21]. Zéboulon et al. also found that using corneal topography raw data such as “elevation against the anterior best fit sphere (BFS), “elevation against posterior BFS”, “axial anterior curvature”, and “pachymetry”, the CNN was able to accurately classify eyes into either “normal”, “KCN”, and “history of refractive surgery” [22]. Askarian et al. also developed a portable, and robust KCN detection method based on smartphone images [23]. Utilising the SVM model, they are able to detect KCN from healthy eyes with an accuracy, specificity and sensitivity of 89%, 91% and 88%, respectively [23].

With eye rubbing being one of the key risk factors of KC development, a group developed a method of detecting frequency and intensity of eye rubbing based on accelerometer and gyroscope data obtainable from wrist-mounted sensor. They were able to analyse these data and detect people with frequent eye rubbing habit using selected ML algorithms such as SVMs and RF with a high precision (precision value = 0.99), and high F1 score of 0.97 [24]. This proposed method is therefore able to accurately detect eye rubbing and consequently serve as an accurate reminder for the user, and thus reduce the risk of KC [24].

### Infectious keratitis

IK refers to an infection of the cornea, or otherwise known as infectious corneal ulcer or opacity [25]. Broadly speaking, it can be caused by various microorganisms including but not limited to bacterial, fungi, parasites, as well as viral agents such as herpes simplex virus (HSV) [26]. Incidence of new cases range from 3.1 to 13.2 per 100,000, with a recurrence rate of 1.2–1.5 times than new cases [27]. A good treatment outcome of IK often relies on timely and accurate diagnosis followed by appropriate interventions i.e., culture-directed therapy. At present, diagnosis is made based on clinical signs and history (positive and negative risk factors), which can be challenging, as IK can be caused by a wide range of organisms mentioned above. This is often complemented by further microbial investigations such as gram stains, culture and sensitivity testing. However, an accurate diagnosis could be delayed due to turnover time especially in fungal keratitis. Other investigation includes molecular diagnostics such as the use of polymerase chain reaction and mass spectrometry which can deliver results quickly but are more costly. As such, clinicians have looked at AI as a potential solution to discriminate between various forms of IK with data from slit-lamp photographs, smartphone photographs, and/or IVCM.

Wang et al. used three CNN models (InceptionV3, ResNet50, DenseNet121) to analyse 5673 slit lamp photographs and 400 smartphone photographs, to detect IK. Amongst the three models, InceptionV3 performed the best (AUC = 0.96) [28]. They also explored if “global image” i.e., an image with additional information beyond the cornea such as sclera, eyelashes, upper and lower eyelids, could allow detection of IK better than just a “regional image” i.e., the cornea [28]. Overall, CNNs, especially Inception V3, were able to detect IK depicted on both slit-lamp photographs and smartphone images, and a model that trained using global images outperformed those that were trained using regional images [28]. This method demonstrated how AI can be used in a primary care setting as it potentially gives primary care physicians or non-ophthalmologists the ability to screen for IK and promptly refer at-risk patients to ophthalmology.

Amongst the non-viral corneal infections, in particular microbial keratitis, the causative agent may vary greatly depending on location, climate, use of contact lens, accessibility to medical care services, and socio-economic status [29]. Specifically, fungi was found to be the most common isolated organism in Asia [30], whereas bacteria are the main culprit in Australia, Europe, and United States of America [31–33]. Fungal causes are usually more severe and require early and appropriate antifungal therapy as these have a higher risk of progressing to endophthalmitis [34]. Ghosh et al. developed a CNN model, namely DeepKeratitis, using a large dataset comprising 2167 eyes that were diagnosed either with bacterial or fungal keratitis [35]. It was found that DeepKeratitis could assist clinicians in rapidly differentiating between the two types with a positive predictive value of 91% and sensitivity of 77% [35].

In addition to slit-lamp photos, IVCM has also been explored in training AI algorithms in detecting fungal keratitis. Liang et al. used a two-streamed network consisting of a mainstream extracting image-level features to extract hierarchical features, and a secondary stream extracting prior-level features and processes corresponding to prior knowledge (such as the approximate structure of hyphae) which might improve classification accuracy [36]. The features from both streams would then be integrated to perform the final prediction. The group found that this method achieved a sensitivity of 97% and specificity 98.5%, with a F1 score of 0.978 [36]. Similarly, Essalat et al. explored the training of CNN models to discriminate acanthamoeba keratitis and fungal keratitis from healthy eyes and other forms of keratitis. DenseNet161 achieved a F1 score of 0.94 for acanthamoeba keratitis and 0.92 for fungal keratitis [37]. IVCM could be used to complement laboratory testing, which are time-consuming. However, interpretation of IVCM could be challenging especially for the untrained [38]. Hence, these models could act as an adjunct for clinicians who are unfamiliar with IVCM to guide their management in suspect cases of acanthamoeba and fungal keratitis.

For viral keratitis such as HSV stromal infection, diagnosis may be difficult as it can mimic bacterial and fungal keratitis. Various CNNs have been trained in identifying HSV stromal necrotising keratitis, namely DenseNet, RestNet, Inception [39]. Overall, DenseNet was found to have an accuracy of 72%, whilst ResNet has a 50% accuracy, and Inception has a 62.5% accuracy. It was thought that whilst the CNN models do not have a particularly high accuracy, it might be used as a screening tool for non-ophthalmologists to assist them in recognising a potential HSV necrotising stromal keratitis and to promptly refer the patient to an ophthalmologist.

### Pterygium

Pterygium, otherwise known as the surfer’s eye, is a cornea condition characterised by a growth of limbal and conjunctival tissue over the adjacent cornea [40]. The prevalence varies but can be as high as 30% depending on population and location [40]. Whilst generally benign, it has the ability to affect visual acuity through its effect on corneal astigmatism, irregularity and high-order aberrations, or when it covers the visual axis [41].

Fang et al. found that DL algorithms were able to detect pterygium, based on slit-lamp photographs as well as smartphone cameras photographs [42]. The group deployed a CNN which extracts features from the slit-lamp photographs. After going through various layers of the CNN, this would produce an output probability of the images which reflected a particular grading of pterygium. The network was then tested against two external sets and was found to be able to detect pterygium quite reliably with a sensitivity of 95.9% to 100% and specificity of 88.3% to 99.0% (AUC = 0.99) for their external test sets [42]. Overall, the DL algorithms were able to perform optimally with regards to sensitivity and specificity, even when applied to smartphone images [42].

The definitive treatment of pterygium is surgery, and the risk of recurrence postoperative is closely related to the extent of pterygium pre-removal [43]. Liu et al. used ResNet Faster Region based CNN (R-CNN) for feature extraction and trained it based on slit-lamp photos. They subsequently tested R-CNN against slit-lamp photos and smartphone photos, demonstrating the ability to detect pterygium on both slit lamp and smartphone photos [44]. Furthermore, the network was able to grade the pterygium with a F1 score of 0.91, sensitivity of 92%, specificity of 98% and AUC of 0.95 [44]. Similarly, Hung et al.'s method also showed high specificity (91.67% to 100%) and sensitivity (80% to 81.67%) in pterygium grading, and was able to predict the recurrence of pterygium post-excision with a high specificity and accuracy (81.8% and 80%, respectively) [45].

### Dry eye disease

DED is a common ocular condition that can affect one’s quality of life with its myriad of symptoms, affecting visual function with significant socio-economic impact [46], and has a prevalence of 5% to 50% amongst the adult population [47]. Given the debilitating nature of DED, various diagnostic tests have been developed so that appropriate treatment can be applied. Examples of these diagnostic tests include Schirmer’s test, fluorescein, Rose Bengal and Lissamine green staining, tears function index, fluorescein clearance test, tear breakup time (TBUT), and etc. [48]. Current tests are however found to be time consuming, invasive, and potentially unreliable as they are subjective and operator dependent [48–50]. AI tools hence offer the potential to provide an objective diagnostic method in diagnosing DED, identification of patients with pre-clinical DED and early provision preventative management.

A ML model by Fineide et al. was able to predict reduced TBUT and differentiate eyes with reduced TBUT with high accuracy [51]. In addition, other clinical features such as ocular surface staining, meibomian gland dropout, blink frequency, osmolarity, meibum quality and symptom score were found to be important predictors for tear film instability [51]. This was consistent with what has been established by the Tear Film and Ocular Society Dry Eye Workshop II [51]. Similarly, Abdelmotaal et al. used a CNN algorithm for developing an automated diagnostic tool of DED based on video keratoscopy, with a high accuracy and AUC of 0.98 [52]. In another study by Chase et al., AS-OCT was instead utilised in a CNN (VGG19 model) and achieved a sensitivity of 86.4%, specificity of 82.4%, and accuracy of 84.6% [53]. In this proposed method, the group specified the extraction features, followed by utilisation of an end-to-end black box for the decision making process. The algorithm developed its own method to differentiate the DED group from healthy eye groups through highlighting the pattern of tear film-corneal epithelium. The group also compared their DL model with existing clinical tests and found that the model was significantly more in tune with a clinician’s diagnosis as compared to Schirmer’s test, corneal fluorescein staining, and conjunctival lissamine green staining, but is not significantly more than TBUT and ocular surface disease index [53].

Another research group was able to innovate a smartphone attachment called “Smart Eye Camera”, which allows for portable recording of ocular surface video under cobalt blue light at 10 times magnification [54]. The model demonstrated high accuracy with AUC of 0.877, sensitivity 77.8% and specificity of 85.7%, offering a potential future of having portable equipment that allows fast and accurate diagnosis of DED [54].

Interferometry is a non-invasive method for visualizing the lipid layer at the surface of the tear film [55]. A study group proposed a method of using an AI model to discriminate DED from healthy eyes using interferometry images, which involves segmentation of the region of interests and using an extract feature based on phylogenetic diversity indexes. This was then followed by classifications through various algorithms such as SVM, RF, Naïve Bayes, Multilayer Perceptron, Random Tree, and radial basis function neural network [56]. It was found that RF classifier gave the best result, reaching an accuracy of over 97% (AUC = 0.99), and a F1 score of 0.97 [56].

Aside from looking at the ocular surface imaging modalities, other important predictors of DED such as blinking patterns like incomplete blinking was also explored [57, 58]. In a study of 100 patients (50 with DED and 50 with healthy eyes) by Zheng et al., blink videos were recorded using Keratography 5 M (K5M; Oculus Optikgeräte GmbH, Wetzlar, Germany) [59]. A U-Net model was then used to extract 30 frames per second (FPS) white light videos, and 8 FPS infrared light videos, which were then utilised to derive blink profiles. It was found that blink videos with 30 FPS have higher accuracy in detecting incomplete blinking which was a sensitive indicator of DED [59]. Another important predictive factor of DED is meibomian gland dysfunction (MGD) which results in the disruption of the tear film lipid layer and increases the tear film evaporation rate [60]. A mask region based convolutional neural network (RCNN) algorithm has been explored in achieving automatic identification of MGD by applying it on analyses of 1878 non-invasive infrared meibography images [61]. It was able to derive the ratio of meibomian gland loss through precise segmentation and identification of conjunctiva and meibomian glands [61]. Similarly, MGD could be identified through AI algorithm based on analysis of IVCM, and differentiates healthy meibomian gland from obstructive or atrophic meibomian gland [62, 63].

### Artificial intelligence for image analysis and segmentation

In addition to using AI algorithms to develop tools for assisting clinicians in screening and/or diagnosing corneal conditions, AI algorithms have also been explored for the use of image analysis of corneal layers, or structures such as corneal nerves. The list of studies that are related to AI-assisted segmentation and analysis of cornea images are found in Table 3.

**Table 3:** A summary table of artificial intelligence (AI) applications in segmentation of corneal endothelium and nerves, in reverse chronological order

Year	Authors	Imaging modality	Sample size (eyes)	Study population	Outcome measures	AI algorithms	Diagnostic performance	Validation model
Corneal endothelium								
2023	Karmakar et al. [75]	Konan CellCheck XL	612	Healthy and diseased eyes	Segmentation of endothelial cells	Mobile-CellNet CNN	Mean absolute error: 4.06%	Hold-out validation
2022	Qu et al. [136]	IVCM	97	Healthy, FECD and corneal endotheliitis eyes	Segmentation of endothelial cells	CNN	PCC: 0.818–0.932	Hold-out validation
2020	Canavesi et al. [77]	GDOCM	10	Eye bank	Segmentation of endothelial cells	CNN	Correlation: 0.91–0.94	Cross validation
2019	Bennett et al. [80]	JDS Uniphase, TOMEY TMS-5	10	Healthy eyes	Evaluation of corneal thickness	CNN	RMSE: 0.045–0.048 Acc: 84.82%–89.26%	Hold-out validation
2019	Vigueras-Guillén et al. [137]	Topcon SP-1P	738	Patients with Baerveldt glaucoma device and DSAEK	Segmentation of endothelial cells	CNN	Mean absolute error: 4.32%–11.74%	Hold-out validation
2019	Daniel et al. [70]	Topcon SP-3000	385	Database of healthy, endothelial disease and corneal graft eyes	Segmentation of endothelial cells	U-Net CNN	PCC: 0.96, Sens: 0.34% Precis: 0.84%	Hold-out validation
2018	Fabijańska et al. [73]	Specular microscopy	30	Dataset of endothelial cell images	Evaluation of corneal thickness	U-Net CNN	AUC: 0.92, Dice: 0.86 Mean absolute error: 4.5%	Hold-out validation
2018	Vigueras-Guillén et al. [76]	Topcon SP-1P	103	Dataset of endothelial cell images	Evaluation of corneal thickness	SVM	Precis: *P* < 0.001 Acc: *P* < 0.001	Cross validation
Corneal nerves								
2023	Li et al. [93]	HRT-3 confocal microscopy	30	Eyes with slight xerophthalmia	Reconstruction of CSNP in images	NerveStitcher CNN	No validation or qualitative evaluation	N.A
2022	Setu et al. [88]	IVCM	197	Healthy and DED eyes	Segmentation of CNF and DC	U-Net, Mask R CNNs	Sens: 86.1%–94.4%, Spec: 90.1% Precis: 89.4%, ICC: 0.85–0.95	Cross validation
2022	Mou et al. [89]	HRT-3 confocal microscopy	300	CORN1500 dataset images	Grading of corneal nerve tortuosity	ImageNet, AuxNet	Acc: 85.64%	Cross validation
2021	Zéboulon et al. [95]	AS-OCT	607	Healthy and edematous corneas	Measurement of edema fraction	CNN	Threshold for diagnosis: 6.8%, AUC: 0.994, Acc: 98.7% Sens: 96.4%, Spec: 100%	Hold-out validation
2021	Deshmukh et al. [96]	ASP	504	Genetically confirmed GCD2 patients	Segmentation of cornea lesions	U-Net, CNN	IoU: 0.81 Acc: 99%	Cross validation
2021	Salahouddin et al. [138]	CCM	534	Healthy and type I diabetic eyes	DPN detection	U-net CNN	κ: 0.86, AUC: 0.86–0.95 Sens: 84%–92%, Spec: 71%–80%	Hold-out validation
2021	McCarron et al. [86]	HRT-3 confocal microscopy	73	Healthy and SIV-infected macaque eyes	Characterize difference in CSNP in acute SIV infection	deepNerve CNN	SIV infection reduced CNFL and fractal dimension (*P* = 0.01, *P* = 0.008)	N.A
2021	Yıldız et al. [139]	HRT-3 confocal microscopy	85	Healthy and chronic ocular surface pathology eyes	Segmentation of CSNP	GAN, U-Net CNN	PCC: 0.847–0.883 AUC: 0.8934–0.9439	N.A
2020	Scarpa et al. [85]	CCM	100	Healthy and DPN eyes	Classification of DPN and healthy eyes	CNN	Acc: 96%	Cross validation
2020	Williams et al. [84]	CCM	2137	Healthy and DPN eyes	Quantification of CSNP, detection of DPN	CNN	ICC: 0.656–0.933, AUC: 0.83 Spec: 87%, Sens: 68%	Hold-out validation
2020	Wei et al. [140]	HRT-3 confocal microscopy	139	Healthy eyes	Segmentation of CSNP	CNS-Net CNN	AUC: 0.96, Precis: 94% Sens: 96%, Spec: 75%	Hold-out validation

*Acc* = accuracy; *ANFIS* = adaptive neurofuzzy inference system; *AS-OCT *= anterior-segment optical coherence tomography; *ASP* = anterior-segment photography; *AUC* = area under curve; *CCM* = corneal confocal microscopy; *CNF* = corneal nerve fibers; *CNFL* = corneal nerve fiber length; *CNN* = convoluted neural networks; *CSNP* = corneal sub-basal nerve plexus; *DC* = dendritic cells; *DED* = dry eye disease; *DPN* = diabetic peripheral neuropathy; *DSAEK* = Descemet stripping automated endothelial keratoplasty; *FECD* = Fuchs endothelial corneal dystrophy; *GDOCM* = Gabor-domain optical coherence microscopy; *GRBF* = Gaussian radial basis function; *HIS* = hyperspectral imaging; *ICC* = interclass correlation coefficient; *IoU* = intersection over union; *IVCM* = in vivo confocal microscopy; *κ* = kappa index; *N.A.* = not available; *PCC* = Pearson’s correlation coefficient; *PEE* = punctate epithelial erosions; *Precis* = precision; *RMSE* = root mean square error; *Sens* = sensitivity; *Spec* = specificity; *SVM* = support vector machine

### Endothelial cell count

The endothelium refers to a layer of polygonal cells which plays an important role in preserving corneal transparency and hydration [64]. Endothelial cell density (ECD), along with polymegathism (or cell variation, CV) and pleomorphism (or hexagonality, HEX) reflect the health and function of endothelium [65]. ECD is found to be the highest at birth which declines to 2500 cells/mm² at adulthood [64]. The minimum ECD required to maintain its function is 400–500 cells/mm², and any value below this could cause corneal edema and reduced visual function [64]. Endothelial cell count or density can be influenced by Fuchs endothelial corneal dystrophy (FECD), ocular surgery, raised intracranial pressure, systemic issues such as diabetes mellitus [64, 66]. ECD is often derived from the use of specular microscopy or IVCM which provides a magnified view of the endothelium [67], and is analyzed via accompanied computer-assisted morphometry [68, 69]. These commercially available software whilst able to perform quantitative analysis of the endothelial images, have limitations as well due to their tendency to under- or over-estimate, especially in advanced endothelial diseases, where the images are of poor quality, leading to under- or over-segmentation of endothelial cells [70]. Various methods have been proposed to circumvent these issues such as the application of directional filtering which can be labor intensive as missing or false cell boundaries are required to be manually edited [71], and watershed algorithms tend to overestimate cells in areas with poor image quality [72].

CNN has been used as a proposed method to automatically segment and analyze specular microscopy images [70, 73]. Fabijańska trained a U-Net algorithm to discriminate pixels located at the borders between cells, and the network would produce an edge probability map which will be binarized and skeletonized to obtain one-pixel wide edges [73]. When tested on a dataset of 30 corneal endothelial images, the AUC was 0.92, with a Dice’s coefficient of 0.86 [73]. Daniel et al. further applied the same proposed method against “real world” specular microscopy images where images obtained may be of poor quality and/or without clear visible endothelial cells as found in advanced FECD [70]. When tested against such images, the algorithm was able to correctly predict ECD and ignore regions where there was poor visibility of ECD (R^2^ = 0.96, Pearson’s correlation). This was further compared to the classical approach based on grayscale morphology and watery shedding. It was found to be superior to the classical approach (*R*^2^ = 0.35, Pearson’s correlation) [70]. When compared to other CNNs such as SegNet, U-Net was found to be superior in its ability to accurately segmenting endothelial cells [74]. Mobile-CellNet was developed by Ranit et al. as an automatic algorithm to estimate ECD based on a hybrid DL model – through the usage of two similar CNN segmentation models trained in isolation. It worked in parallel with classical image post-processing techniques. This was compared to U-Net and U-Net +  + [75]. Overall, Mobile-CellNet was able to achieve a mean absolute error of 4.06% for ECD, CV and HEX, and was comparable with U-Net (3.80%) [75].

Another proposed method is through the use of SVM algorithms, which start with over-segmented images [76]. The SVM algorithm would then utilize intensity and shape information of super-pixels to merge those that would constitute a single cell. This proposed method achieved a significantly better precision in all parameters (ECD, CV, and HEX) when compared to the commercially available software (*P* < 0.001, *P* = 0.02, *P* < 0.001, respectively) [76].

Gabor-domain optical coherence microscopy (GD-OCM) is an emerging modality which incorporates concepts of optical coherence tomography and confocal microscopy. GD-OCM was found to be able to segment and ECD as well as current standard of practice—specular microscopy [77]. When used in isolation, GD-OCM is able to produce results comparable to that of specular microscopy. The accuracy of GD-OCM could then be further enhanced using CNNs such as U-Net, which was found to be comparable with current practice of analysis using specular microscopy [77].

With an increasing prevalence of laser refractive surgery, there has been a rising need in detecting mild or subclinical forms of ectatic cornea disease due to their higher risk of developing cornea ectasia post-surgery. Recognizing the importance of cornea structure and its biomechanical property as a predictor of cornea ectasia post-surgery, Ambrosio et al. developed a RF algorithm using Scheimpflug images and biomechanical index [78]. The model could reliably detect ectasia with an AUC of 0.996 [78].

Apart from the endothelium, the cornea epithelium could be differentiated, with injuries detected with hyperspectral imaging images processed by CNN (SVM with Gaussian radial basis function, CNN only, and combined SVM-linear and CNNs), without the need of eye staining [79]. CNN and combined SVM-linear and CNN models alone are able to achieve up to 100% accuracy [79]. Corneal thickness can also be measured using an automated process via CNN, which involves a secondary speckle tracking (tracking of a laser beam speckle pattern backscattered from corneal-scleral border) and processing of the obtained data by a CNN [80]. This method was reliable and accurate with a 26 μm of mean fit error, which is comparable to existing pachymetry tools [80].

Diabetic peripheral neuropathy is one of the most prevalent complications of diabetes mellitus, and is found in more than 50% of diabetic patients [81]. In patients with diabetic peripheral neuropathy, there is a progressive loss of corneal nerve fiber, which is a layer of unmyelinated nerve fiber that is local sub-basally [82]. Measurement of the corneal nerve fiber can therefore be used as a form of adjunct assessment in the detection and monitoring of diabetic neuropathy, which typically is performed using IVCM [82, 83]. Multiple studies have explored the use of AI-driven algorithms, in particular CNNs, in assessing IVCM of sub-basal corneal nerves, and was found to have high accuracy and precision [84–87]. CNNs (ResNet and U-Net) can also be used in segmenting and evaluating dendritic cells in addition to sub-basal nerve based on IVCM [88]. A dedicated CNN-algorithm, DeepGrading, was developed by Mou et al. for segmenting and quantifying corneal nerve tortuosity, which demonstrated superiority to existing methods in tortuosity grading, and achieved an accuracy of 85.64% in four-level classifications [89]. IVCM typically provides a 2-dimensional image of the cornea. A montage of IVCM images is therefore imperative to accurately present the status of corneal nerves as it usually runs converging toward an area approximately 1 to 2 mm inferior to the corneal apex in a whorl-like pattern [90].

A deep-learning algorithm, NerveSticher was proposed by Li et al. which uses montages of existing IVCM images and subsequent analysis through the use of a neural network. When compared to existing non-AI driven methods [90–92], it demonstrated superiority in matching accuracy and reduced processing time [93]. Overall, current studies have provided an insight into the future of how DL algorithms such as CNN can provide rapid and excellent performance in segmenting and quantifying cornea nerves, serving as a biomarker in the screening of diabetic peripheral neuropathy in the community.

To understand non-specific ocular pain better, Kundu et al. developed a RF-based algorithm to analyze various cornea nerve parameters, presence or absence of systemic and orthoptic issues, and its relationship with various clinical and imaging parameters such as IVCM, presence of microneuromas, dendritic cells, and etc. [94] They found that the model was robust (AUC of 0.86 and F1 score of 0.86), with the most useful parameters being the presence of microneuromas, immature and mature dendritic cells, presence of orthoptics issues, and nerve fractal dimension parameters [94].

AI-driven tools were also able to segment AS-OCT and detect corneal edema through the use of CNN algorithm and classify them as either “normal” or “edema” [85]. It was found that the mean edema fraction (EF) of a normal eye was 0.0087 ± 0.01 and 0.805 ± 0.26 for edema eyes (*P* < 0.0001), with an AUC of 0.99 [95]. Furthermore, the CNN algorithm was able to produce a corresponding heatmap of the cornea edema [95]. CNN algorithms could also be used in automated segmentation of corneal stromal deposits and is comparable to manual segmentation [96].

### Artificial intelligence in corneal transplantation

Age-related endothelial diseases such as FECD represent one of the leading causes of blindness in the world [97], and can lead to decreasing ECD, with resultant corneal edema and ultimately visual decompensation. The definitive treatment would be a corneal transplant (selective endothelial keratoplasty), such as Descemet stripping automated endothelial keratoplasty (DSAEK) or Descemet’s membrane endothelial keratoplasty (DMEK). The aim is to improve visual function [98]. The list of studies that have described the use of AI-related algorithms in corneal transplants are summarized in Table 4.

**Table 4:** A summary table of artificial intelligence (AI) applications in corneal transplant, in reverse chronological order

Year	Authors	Imaging modality	Sample size (eyes)	Study population	Outcome measures	AI algorithms	Diagnostic performance	Validation model
Corneal transplants								
2023	Hayashi et al. [104]	AS-OCT	300	Patients undergoing DMEK	Predict graft detachment and rebubbling	EfficientNet	AUC: 0.875, Sens: 78.9% Spec: 78.6%	Hold-out validation
2023	Patefield et al. [105]	AS-OCT	24	Patients undergoing DMEK	Predict graft detachment	ResNet-101	Acc: 77%, Precis: 67% Spec: 45%, Sens: 92% AUC: 0.63	Hold-out validation
2022	Bitton et al. [141]	AS-OCT	290	Healthy and FECD pre-DMEK eyes	Corneal edema detection	U-Net models	AUC: 0.97	N.A
2022	Mujizer et al. [142]	91 different parameters	3647	Patients undergoing PLK	Predict graft detachment	Logistic regression, CTA and RF	AUC: 0.65–0.72	Cross validation
2021	Hayashi et al. [106]	AS-OCT	46	Patients undergoing DALK	Predicting success of big bubble formation	VGG16 CNN	AUC: 0.746	Cross validation
2020	Yousefi et al. [99]	CASIA AS-OCT	3162	Post-surgery of PKP, LKP, DALK, DSAEK or DMEK	Predicting the need for future keratoplasty surgery	Unsupervised machine learning	No validation of prediction	N.A
2020	Hayashi et al. [143]	AS-OCT	31	Patients post-DMEK requiring and not requiring rebubbling	Predicting the need for rebubbling post-DMEK	Multiple CNNs	AUC: 0.964, Sens: 96.7% Spec: 91.5%	N.A
2020	Heslinga et al. [103]	AS-OCT	1280	Patients post-DMEK	Localize and quantify graft detachment	CNN	Dice: 0.896	Hold-out validation
2019	Treder et al. [144]	AS-OCT	1172	Patients post-DMEK	Detect graft detachment	Classifier tree	Acc: 96%, Sens: 98% Spec: 94%	Hold-out validation

*Acc* = accuracy; *AS-OCT* = anterior-segment optical coherence tomography; *AUC* = area under curve; *CNN* = convoluted neural networks; *CTA* = classification tree analysis; *DALK* = deep anterior lamellar keratoplasty; *DMEK* = Descemet membrane endothelial keratoplasty; *DSAEK* = Descemet stripping automated endothelial keratoplasty; *FECD* = Fuchs endothelial corneal dystrophy; *LKP* = lamellar keratoplasty; *PKP* = penetrating keratoplasty; *N.A*. = not available; *PLK* = posterior lamellar keratoplasty; *Precis* = precision; *RF* = random forest; *Sens* = sensitivity; *Spec* = specificity

To develop a screening tool for surgeons to screen for patients who are likely to require cornea transplant, Yousefi et al. utilized an unsupervised ML to identify corneal conditions and predict the likelihood for future keratoplasty based on AS-OCT features [99]. This promises to be an objective tool for surgeons to identify patients who, as a result of increased risk of future keratoplasty, require closer monitoring.

Partial graft detachment is a common complication post-DMEK [100]. Clinical management may differ based on the surgeon’s clinical evaluation and experience [101]. AS-OCT has been commonly used by cornea surgeons to guide decision for re-bubbling as it allows direct visualization of graft detachment. Quantification of graft detachment can be challenging due to several factors: 1) AS-OCT consists of multiple radial B-scan which must be interpreted for a good overview of the condition, and 2) the regions where the graft is appositioned to has yet to be attached, and may masquerade as graft detachment [102, 103]. A CNN trained by Heslinga et al. used 80 scans to automatically locate scleral spur and quantify the degree of graft detachment [103]. The proposed method has a high accuracy with the mean scleral spur localization being 0.155 mm, and when compared to manually segmented images, has a Dice coefficient of 0.88 to 0.90 [103]. By providing an objective measure such as measurement of the degree of graft detachment, it allows a classification system to be developed in the future which can help guide surgeons’ management dilemmas, such as if the patient is for re-bubbling or for observation (and its follow-up intervals).

Beyond segmenting and quantifying the degree of graft detachment, effort has also been put into developing a tool to predict graft detachment [104, 105]. Patefield et al. used ResNet-101, a CNN, to train on AS-OCT B-scans obtained from 50 eyes, which was then further validated and tested on scans obtained from 24 eyes [105]. In essence, the proposed model has a relatively high accuracy with a F1 score of 0.77 (AUC = 0.63), high sensitivity (92%), and low specificity (45%) [105]. It was found to have better a predictive level when compared with a seasoned clinician who predicted based on clinical information and interpretation of AS-OCT images (sensitivity of 92% *vs.* 31%) [105]. Similarly, Hayashi et al. proposed a similar method using EfficientNet to predict the probability of graft detachment and by extension, the probability of re-bubbling needed based on AS-OCT images pre-operation, with an AUC of 0.88, sensitivity of 78.9% and specificity of 78.6% [104]. The DL algorithm was able to identify and extract features from AS-OCT images, to extrapolate the potential risk of graft detachments and re-bubbling post-operation, allowing clinicians to better prognosticate and counsel their patients for the decision of operation.

Havashi et al. also looked at the use of a DL network (VGG16) to predict the probability of successful big-bubble formation during deep anterior lamellar keratoplasty (DALK), which is a common procedure for KC and other conditions that could cause corneal opacification [106]. This was based on pre-operative images of AS-OCT and other cornea biometric parameters [106]. The prediction success rate was 78.3% for big bubble formations, and 69.6% for failed big bubble formation (AUC = 0.75) [106]. It was also found that eyes with KC are of higher likelihood to have successful big-bubble formation, compared to non-KC eyes undergoing DALK [106].

### Open access cornea data

With the uprising of development in AI-based tool within the realm of cornea diseases, although limited, there exist some open access cornea image datasets and/or parameters which allow other study groups to test their own AI models against.

Such examples include CORN 1, a nerve segmentation dataset from BioImLab, University of Padova (https://github.com/iMED-Lab/CS-Net), CORN 2, a confocal image enhancement dataset, and CORN 3, a nerve tortuosity estimation dataset. The latter two require application to the lab for use. Cai et al. also developed and provided a large scale anterior eye segment photos dataset called EyeHealer, with both eye structure and lesions being annotated at pixel level, to also allow other groups to test their AI tools on [107].

### Current challenges and future of artificial intelligence for cornea

In the near future, there will be more research surrounding the use of ML and/or DL algorithms in the field of cornea to enhance 1) diagnostic performance, 2) segmentation and analysis of images to allow better monitoring of diseases, and 3) prediction of outcomes of surgery (such as corneal transplant). Overall, the performance of most methods described above seem to be favorable as most are often accurate and either comparable or superior to its counterpart non-automated assessment.

AI in Ophthalmology, and specifically applied to the cornea, is still at its infancy stage. One of the key challenges in the AI field is the issue with “AI black box”, which leads to an ethical dilemma as decision processes adopted by the algorithm are neither known to us, nor is tracked by the DL network itself. Whilst assessments made by DL networks are objective, its reliability and accuracy rely on the training dataset, which could be of poor quality. In particular, development of an AI-based image analysis/segmentation tool often relies heavily on edge detection method which could be sensitive to noise and inaccurate detection. It may also require manual tuning depending on the method used. This may lead to the development of algorithms that make inaccurate or inconsistent decisions, leading to potential medico-legal issues as well as impairing the quality of care delivered. Furthermore, management plans for patients with cornea conditions should be individualized and tailored according to their circumstances such as quality of life, premorbid, financial situations, and etc. Therefore, whilst an algorithm-based system may assist in detecting and predicting the progression of the disease, a clinician is still needed to use information from the AI model together with other factors to provide the best individualized care. Another issue with the AI-based system as identified by Li et al. is its longevity due to the ever changing profile of diseases, population, healthcare infrastructure, and technologies [108]. There also exist AI bias as a result of differences in software or hardware differences, population and ethnicities, generalizability, privacy, and etc. [109].

Some research groups may have attempted to validate with “real world” images which are of poor quality, poorly segmented or compounded by co-ocular conditions, but most algorithms were trained with small sample sizes and validated against internal datasets of pre-selected images. The method of validation also differs from group to group, with some conducting hold-out validation and others cross-validation, making direct comparison of results difficult. Furthermore, reporting bias exists, with trained algorithms with unfavorable outcomes or performances potentially not being reported. Most methods are also experimental and have yet to be tested in a large clinical trial, and hence merely remains an area of interest in the realm of research which has yet to be adopted in clinical practice.

Although the Consolidated Standards of Reporting Trials-AI (CONSORT-AI) and Standard Protocol Items: Recommendations for Interventional Trials-AI (SPIRIT-AI), were introduced to allow for an international-based reporting guidelines for AI-related studies, these were only introduced in recent years [110]. Prior to those, there was no uniformed structure in reporting of AI-related studies, making comparison of studies difficult. Researchers should continue to publish studies in accordance with these guidelines to allow clinicians, patients, and authorities to be able to evaluate and compare the efficacy of AI-related studies. Furthermore, while there are open access library of ML and DLN code currently available online for various groups to adopt and use in their research, we found that there are limited availability of open access pre-trained networks specifically for the cornea. This is likely related to the fact that this area is still in its infancy stage where various groups are in the process of developing and testing their tools. As we progress, an open access library should be developed such that various algorithms can be tested and utilized on images from different populations and establish their usefulness in a clinical setting.

## Conclusion

Artificial intelligence has emerged as a useful adjunctive tool for clinicians as either an assistive, triaging or detection tool for various cornea diseases, or enhancing cornea imaging through efficient analyses. However, this field is still in its infant stage with various barriers to implementation in the clinic. Nonetheless, rapid improvements with real-world data studies have emerged recently, which may lead to clinical applications that could benefit cornea specialists to improve clinical and surgical outcomes in the near future.

### Supplementary Information

**Additional file 1: Table S1.** A summary table of artificial intelligence (AI) applications in keratoconus, from before year 2022, in reverse chronological order.

## Data Availability

Not applicable.

## Abbreviations

AI: Artificial intelligence
AUC: Area under curve
AS-OCT: Anterior-segment optical coherence tomography
BFS: Best fit sphere
CNN: Convolutional neural network
CONSORT-AI: Consolidated standard of reporting trials-artificial intelligence
CV: Cell variation
DALK: Deep anterior lamellar keratoplasty
DED: Dry eye disease
DL: Deep learning
DMEK: Descemet’s membrane endothelial keratoplasty
DSAEK: Descemet stripping automated endothelial keratoplasty
ECD: Endothelial cell density
EDTL: Ensemble of deep transfer learning
FECD: Fuchs endothelial corneal dystrophy
FFKC: Forme fruste keratoconus
FPS: Frames per second
GD-OCM: Gabor-domain optical coherence microscopy
HEX: Hexagonality
HSV: Herpes simplex virus
IK: Infective keratitis
IVCM: In vivo confocal microscopy
KC: Keratoconus
KCS: Keratoconus suspect
MGD: Meibomian gland dysfunction
ML: Machine learning
NN: Neural network
RCNN: Region based convolutional neural network
RF: Random forest
RNN: Recurrent neural network
SD-OCT: Spectral-domain optical coherence tomography
SPIRIT-AI: Standard protocol items: recommendations for interventional trials-artificial intelligence
SKC: Subclinical keratoconus
SVM: Support vector machine
TBUT: Tear breakup time
