# Supplementary material for: Potential applications of artificial intelligence in image analysis in cornea diseases: a review
Source: Eye Vis (Lond). 2024 Mar 7;11:10. doi: 10.1186/s40662-024-00376-3 (PMC10919022; doi:10.1186/s40662-024-00376-3)
Supplement: Supplementary file 1 — Additional file 1: Table S1. A summary table of artificial intelligence (AI) applications in keratoconus, from before year 2022, in reverse chronological order. [file 40662_2024_376_MOESM1_ESM.docx]

**Table S1**: A summary table of artificial intelligence (AI) applications in keratoconus, from before year 2022, in reverse chronological order.

| **Year** | **Authors** | **Imaging modality** | **Sample size (eyes)** | **Study population** | **Outcome measures** | **AI algorithms** | **Diagnostic performance** | **Validation model** |
| --- | --- | --- | --- | --- | --- | --- | --- | --- |
| **Keratoconus** | | | | | | | | |
| 2021 | Ghaderi et al. [146] | Pentacam | 450 | Healthy and KC eyes | KC detection and grading | Ensemble classifier, MLP NFS, NBC | Acc: 98.2%, Sens: 98.5%–99.1%  Spec: 96.2%–99.4% | Hold-out validation |
| 2021 | Al-Timemy et al. [147] | Pentacam | 542 | Healthy, suspect and KC eyes | KC detection and grading | Hybrid deep learning | AUC: 0.93–0.99 | Hold-out validation |
| 2021 | Cao et al. [13] | Pentacam | 167 | Healthy and subclinical KC eyes | Subclinical KC detection | RF | Acc: 98%, Sens: 97% Spec: 98% | Cross validation |
| 2021 | Aatila et al. [148] | CASIA AS-OCT | 12,242 | Healthy and KC eyes | KC grading | SFS RF | Acc: 95%–98% | Cross validation |
| 2021 | Feng et al. [14] | Pentacam | 854 | Healthy, subclinical and KC eyes | Subclinical and KC detection | KerNet CNN | Acc: 98.25%, Sens: 93.71% Precis: 94.10%, κ: 0.918 | Cross validation |
| 2020 | Abdelmotaal et al. [149] | Pentacam | 3,218 | Healthy, subclinical and KC eyes | KC detection and grading | CNN | Acc: 95.8% | Hold-out validation |
| 2020 | Kuo et al. [11] | TMS-4 | 206 | Healthy and subclinical KC eyes | KC detection | CNN | Acc: 93.1%–95.8%, Sens: 91.7%–94.4% Spec: 94.4%–97.2%, ROC: 0.956–0.995 | Hold-out validation |
| 2019 | Lavric et al. [150] | Pentacam | 1,350 | Healthy and KC eyes | KC detection | KeratoDetect CNN | Acc: 99.3% | Hold-out validation |
| 2019 | Issarti et al. [151] | Pentacam | 838 | Healthy, suspect and KC eyes | KC detection | FNN | Acc: 96.6%, Sens: 95.6% Spec: 97.8% | Cross validation and hold-out validation |
| 2019 | Dos Santos et al. [152] | Pentacam | 20,160 | Healthy and KC eyes | KC detection | CNN | Acc: 99.6% Sens: 99.4% | Hold-out validation |
| 2019 | Kamiya et al. [153] | UHR-OCT | 304 | Healthy and grade 1-4 KC eyes | KC detection and grading | CNN | Acc: 99.1%, Sens: 98.4% Spec: 100% | Cross validation |
| 2018 | Yousefi et al. [16] | CASIA AS-OCT | 3,156 | Healthy and grade 1-4 KC eyes | KC detection and grading | Unsupervised machine learning | Sens: 97.7% Spec: 94.1% | N.A. |
| 2017 | Hidalgo et al. [154] | Pentacam | 131 | Healthy, suspect and KC eyes | KC detection | SVM and binary classification | Acc: 98.9% Spec: 99.1% | Cross validation |
| 2017 | Ambrósio et al. [78] | Pentacam  Oculus Corvis ST | 950 | Health and KC eyes | Ectasia detection | Logistic regression analysis with forward stepwise inclusion, SVM, and RF | AUC: 0.996  Sens: 100%  Spec: 100% | Cross validation |
| 2016 | Hidalgo et al. [155] | Pentacam | 860 | Healthy, FF and KC eyes | KC detection | SVM and binary classification | Acc: 98.9%, Sens: 99.1% Spec: 98.5% | Cross validation |
| 2014 | Silverman et al. [156] | Artemis-1 | 294 | Healthy and KC eyes | KC detection | CNN | AUC: 1.00, Sens: 98.9% Spec: 99.5% | Hold-out validation |
| 2013 | Smadja et al. [157] | Galilei | 372 | Healthy, FF and KC eyes | KC detection | Classification tree | Sens: 99.5% Spec: 100% | Cross validation |
| 2012 | Arbelaez et al. [158] | Sirius | 3,502 | Healthy, FF, KC and post-corneal surgery eyes | KC detection | SVM | Acc: 98.2%, Sens: 95.0% Spec: 99.3% | Hold-out validation |
| 2010 | Souza et al. [159] | Orbscan II | 318 | Healthy, KC and post-refractive surgery eyes | KC detection | RBFNN, SVM, MLP | AUC: 0.98–0.99. Sens: 100% Spec: 98.0% | Cross validation |
| 2005 | Twa et al. [160] | Keratron | 244 | Healthy and KC eyes | KC detection | Decision tree | Acc: 93%, Sens: 93% Spec: 92% | Cross validation |
| 2002 | Accardo et al. [161] | EyeSys | 396 | Healthy and KC eyes | KC detection | CNN | Acc: 96.7%, Sens: 94.1% Spec: 97.6% | Hold-out validation |
| 2000 | Chastang et al. [162] | EyeSys | 208 | Healthy, KC, post-PKP and post-refractive surgery eyes | KC detection | Decision tree | Sens: 94.9% Spec: 88.5% | Hold-out validation |
| 1997 | Smolek et al. [10] | TMS-1 | 300 | KC and KC suspect | KC detection | CNN | Acc: 100%, Sens: 100% Spec: 100% | Hold-out validation |

Acc = accuracy; AS-OCT = anterior-segment optical coherence tomography; AUC = area under curve; CNN = convoluted neural networks; FF = forme fruste keratoconus; FNN = feedforward neural network; κ = kappa index; KC = keratoconus; MLP = multilayer perceptron; N.A. = not available; NBC = naïve Bayes classifier; NFS = neurofuzzy system; PKP = penetrating keratoplasty; Precis = precision; RBFNN = radial basis function neural network; RF = random forest; Sens = sensitivity; SFS = sequential forward selection; Spec = specificity; SVM = support vector machines; UHR-OCT = ultrahigh resolution optical coherence tomography
